# Investigation of WQ-3810, a Fluoroquinolone with a High Potential Against Fluoroquinolone-Resistant *Mycobacterium avium*

**DOI:** 10.3390/antibiotics14070704

**Published:** 2025-07-14

**Authors:** Sasini Jayaweera, Pondpan Suwanthada, David Atomanyi Barnes, Charlotte Poussier, Tomoyasu Nishimura, Naoki Hasegawa, Yukiko Nishiuchi, Jeewan Thapa, Stephen V. Gordon, Hyun Kim, Chie Nakajima, Yasuhiko Suzuki

**Affiliations:** 1 Division of Bioresources, Hokkaido University International Institute for Zoonosis Control, Sapporo 001-0020, Japan; sasini@czc.hokudai.ac.jp (S.J.); dbarnes@czc.hokudai.ac.jp (D.A.B.);; 2 Keio University Health Center, Yokohama 223-8521, Japan; 3 Department of Infectious Diseases, Keio University School of Medicine, Tokyo 160-8582, Japan; 4 Toneyama Institute for Tuberculosis Research, Osaka City University Medical School, Toyonaka 560-8552, Japan; 5 Microbial Genomics and Ecology, The IDEC Institute, Hiroshima University, Higashi-Hiroshima 739-8530, Japan; 6 School of Veterinary Medicine, University College Dublin, D04 V1W8 Dublin, Ireland; stephen.gordon@ucd.ie; 7 Institute for Vaccine Research and Development (IVReD), Hokkaido University, Sapporo 001-0021, Japan; 8 Department of Bacteriology II, Japan Institute for Health Security (JIHS), Tokyo 208-0011, Japan

**Keywords:** Fluoroquinolone resistance, *Mycobacterium avium*, DNA gyrase, supercoiling inhibitory assay, minimum inhibitory concentration, checkerboard assay

## Abstract

**Background/Objectives:** *Mycobacterium avium*, a member of *Mycobacterium avium* complex (MAC), is an emerging opportunistic pathogen causing MAC-pulmonary disease (PD). Fluoroquinolones (FQs), along with ethambutol (EMB) and rifampicin, are recommended for macrolide-resistant MAC-PD; however, FQ-resistant *M. avium* have been reported worldwide. WQ-3810 is an FQ with high potency against FQ-resistant pathogens; however, its activity against *M. avium* has not yet been studied. **Methods**: In this study, we conducted a DNA supercoiling inhibitory assay to evaluate the inhibitory effect of WQ-3810 on recombinant wild-type (WT) and four mutant DNA gyrases of *M. avium* and compared the IC_50_s of WQ-3810 with those of ciprofloxacin (CIP), levofloxacin (LVX), and moxifloxacin (MXF). In addition, we examined WQ-3810’s antimicrobial activity against 11 *M. avium* clinical isolates, including FQ-resistant isolates, with that of other FQs. Furthermore, we assessed the synergistic action of WQ-3810 with the combination of either EMB or isoniazid (INH). **Results**: In a DNA supercoiling inhibitory assay, WQ-3810 showed 1.8 to 13.7-fold higher efficacy than LVX and CIP. In the MIC assay, WQ-3810 showed 4 to 8-fold, 2 to 16-fold, and 2 to 4-fold higher antimicrobial activity against FQ-resistant isolates than CIP, LVX, and MXF, respectively. The combination of WQ-3810 and INH exhibited a synergistic relationship. **Conclusions**: The overall characteristics of WQ-3810 demonstrated greater effectiveness than three other FQs, suggesting that it is a promising option for treating FQ-resistant *M. avium* infections.

## 1. Introduction

*M. avium* is a slow-growing, acid-fast, opportunistic intracellular pathogen belonging to the *Mycobacterium avium* complex (MAC) and nontuberculosis mycobacteria (NTM) [1,2,3]. *M. avium* is widely distributed and can be found in both natural and domestic settings, such as soil, water, and plants, as well as in showerheads and plumbing systems [2,4,5]. The most prevalent clinical manifestation of MAC infection is pulmonary disease (MAC-PD), especially found in immunocompromised individuals, such as those with HIV, and immunocompetent patients with chronic pulmonary diseases such as chronic obstructive pulmonary disease (COPD), cystic fibrosis, and previous pulmonary tuberculosis [1,5]. Previous studies indicate that the prevalence of MAC-PD is rising, with increasing cases being documented in North America, Oceania, Europe, Japan, South Korea, and elsewhere [3,6,7]. The American Thoracic Society guidelines suggest a macrolide-based multidrug regimen for the treatment of MAC-PD until a negative culture is achieved, followed by an additional year of therapy [8,9,10]. However, previous studies have shown that 20–40% of patients do not respond to the combination of three drugs: rifampicin, ethambutol, and macrolide [3,8,9,10,11]. For individuals whose MAC infection is resistant to first-line drugs or who are intolerant of them, a daily dosage of 400 mg of moxifloxacin (MXF) is recommended [8,11,12]. Moreover, a previous study indicated that fluoroquinolones (FQs) were prescribed for the treatment of MAC-PD in 45% of patients in European countries and 71% of patients in Japan [13]. Hence, the introduction of a novel drug with significant efficacy for treating FQ-resistant MAC is essential.

FQ, a synthetic antibiotic, has a broad-spectrum antibacterial activity that inhibits the activity of DNA gyrase [14]. DNA gyrase is a type II DNA topoisomerase, composed of two proteins; subunit A (GyrA) and subunit B (GyrB), that play a crucial role in bacterial DNA transcription and replication by adding negative supercoiling to DNA [15,16]. DNA gyrase covalently binds to double-stranded DNA and breaks it, unwinding the kinks and resealing the DNA. Quinolones bind to the cleaved ends, stabilizing the gyrase-DNA cleavage complex and preventing DNA resealing, thereby killing the bacteria [15]. Previous studies indicate that amino acids at positions 91 and 95 in the quinolone resistance-determining region (QRDR) of GyrA are essential for FQ binding, and amino acid substitutions at those positions lead to FQ-resistant *M. avium* [17,18,19]. In Japan, highly FQ-resistant *M. avium* clinical isolates with GyrA-Asp95Gly and GyrA-Asp95Tyr substitutions have been reported [17].

WQ-3810 is a newly developed FQ which has high lipophilicity, membrane permeability, good oral absorption, and low adverse effects [20]. It shows high efficacy against multi-drug resistant and FQ-resistant Gram-negative bacteria such as *Escherichia coli*, *Acinetobacter baumannii,* and Gram-positive bacteria such as *Streptococcus pneumonia* and methicillin-resistant *Staphylococcus aureus* [21]. According to recent studies, WQ-3810 showed high antimicrobial activity and high enzyme inhibitory activity against *Campylobacter jejuni, M. tuberculosis,* and *M. leprae* [22,23,24]. However, the efficacy of WQ-3810 against FQ-resistant *M. avium* has not been evaluated. Therefore, this study aims to evaluate the inhibitory effect of WQ-3810 on DNA gyrase against FQ-resistant *M. avium* using recombinant *M. avium* wild-type (WT) and mutant DNA gyrases, as well as the antimicrobial activity and the synergistic relationship of WQ-3810 with cell wall inhibitors on clinical isolates of *M. avium*.

## 2. Results

### 2.1. Inhibitory Effects of WQ-3810 Against M. avium WT and Mutant DNA Gyrases

**Figure 1:**
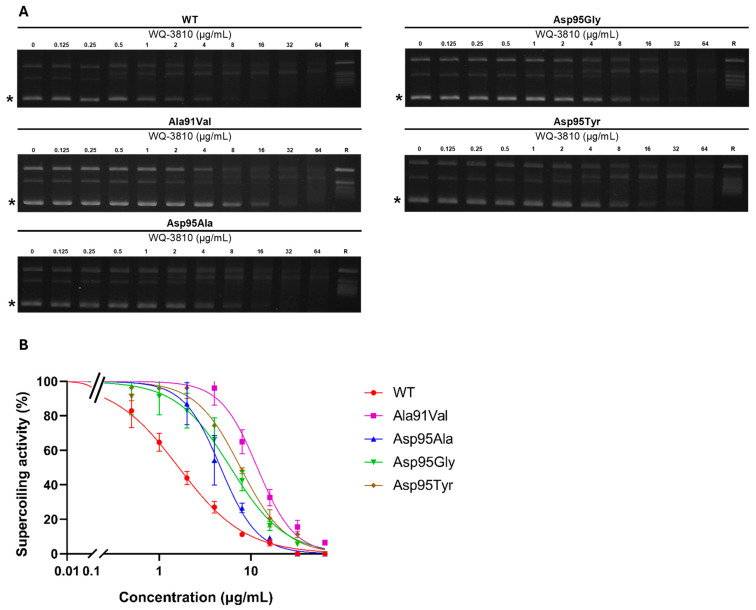
Inhibition of DNA supercoiling activity of *M. avium* DNA gyrase by WQ-3810. (**A**) Representative gel electrophoresis: Gel electrophoresis results demonstrate the separation of supercoiled DNA from relaxed DNA. Supercoiled DNA bands are indicated by asterisks (*). These figures are randomly selected from a triplicate assay. (**B**) Quantification of supercoiled DNA: The levels of supercoiled DNA produced in the presence of WQ-3810 were quantified.

The gel electrophoresis patterns shown in Figure 1 demonstrate a concentration-dependent reduction in the intensity of the supercoiled DNA band, indicating a dose-dependent inhibition of DNA gyrase activity by WQ-3810.

The concentrations of WQ-3810 that inhibits 50% of DNA gyrase activity (IC_50_) for four mutant DNA gyrases were higher than that for WT DNA gyrase (Table 1). The GyrA-Ala91Val mutant exhibited the highest IC_50_ of 9.9 µg/mL, while GyrA-Asp95Ala had the lowest IC_50_ at 4.5 µg/mL. The IC_50_s of the GyrA-Asp95Gly mutant (7.0 µg/mL) and of the GyrA-Asp95Tyr mutant (7.8 µg/mL) demonstrated comparable resistance.

**Figure 2:**
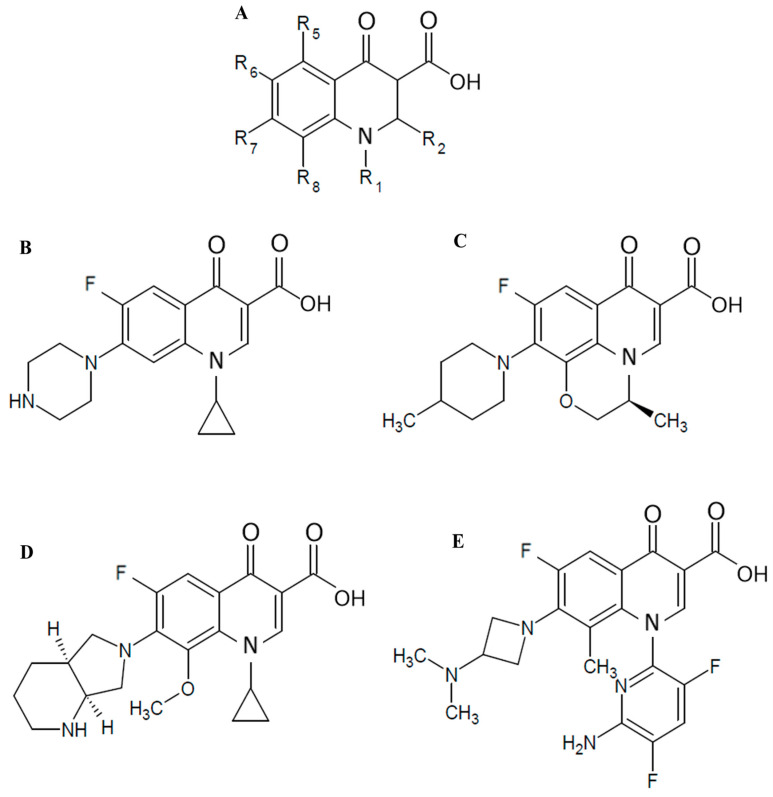
Structures of FQs used in this study. (**A**) The basic structure of FQ. (**B**) CIP (**C**) LVX (**D**) MXF (**E**) WQ-3810.

**Figure 3:**
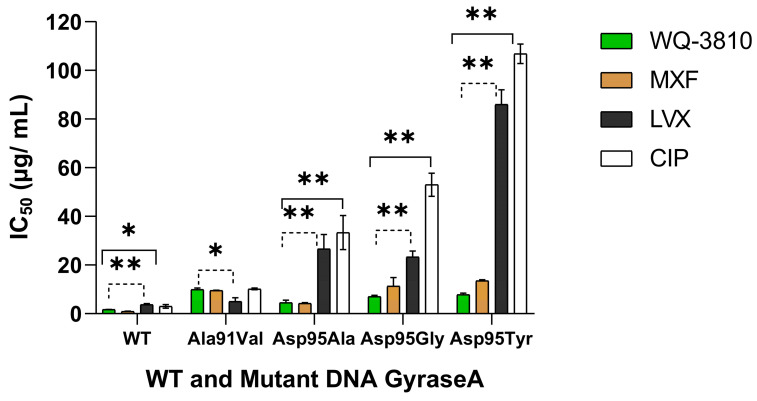
FQ activities against *M. avium* DNA gyrase. The amount of DNA supercoiled by DNA gyrase was measured using agarose gel electrophoresis in triplicate, and IC_50_ was calculated. The normality of the data was tested using the Shapiro–Wilk test. One-way ANOVA was used to compare the differences between the FQs. The least significant difference (LSD) test was used to identify the groups whose means were statistically significantly different. (*, *p* < 0.05, **, *p* < 0.001).

Then, we compared the inhibition results of WQ-3810 with those of other FQs, namely ciprofloxacin (CIP), levofloxacin (LVX), and moxifloxacin (MXF) (Table 1 and Table 2 and Figure 2) [17]. For GyrA-WT, MXF and WQ-3810 showed comparable low IC_50_s of 0.9 µg/mL and 1.7 µg/mL, respectively, while CIP and LVX showed higher IC_50_s (*p* < 0.05) (Table 1, Figure 3). CIP demonstrated the highest IC_50_s for all mutants. For GyrA-Asp95 mutants, IC_50_s of CIP and LVX were significantly higher than those of MXF and WQ-3810 (*p* < 0.001). Although the difference between MXF and WQ-3810 was not significant, IC_50_s of WQ-3810 for GyrA-Asp95Gly and GyrA-Asp95Tyr (7.0 µg/mL and 7.8 µg/mL, respectively) were lower than those of MXF (11.3 µg/mL and 13.5 µg/mL, respectively).

### 2.2. Minimum Inhibitory Concentration (MIC) of WQ-3810

Table 3 shows that WT isolates had significantly lower MICs for all four FQs, ranging from 0.125 to 4 µg/mL, compared to mutant isolates that had MICs from 4 to 128 µg/mL. The MIC ranges for WQ-3810 and MXF were 0.125–1 µg/mL and 0.25–1 µg/mL, respectively, in WT isolates. Furthermore, the study found higher MICs for GyrA-Asp95Gly and GyrA-Asp95Tyr in LVX and CIP than in MXF and WQ-3810. Notably, the MICs of MXF, ranging from 8 to 16 µg/mL for GyrA-Asp95Gly and GyrA-Asp95Tyr, respectively, were found to be higher than those of WQ-3810, which ranged from 4 to 8 µg/mL.

### 2.3. Effects of Cell Wall Synthesis Inhibitors on the MIC of WQ-3810

We used the combination of 1 µg/mL of EMB and INH with MXF and WQ-3810 given in Table 3 to determine the effects of cell wall synthesis inhibitors on the MIC of MXF and WQ-3810. MXF and WQ-3810 yielded comparable or lower MIC results when used together with either 1 µg/mL EMB or 1 µg/mL INH for WT. WQ-3810 in combination with 1 µg/mL EMB or 1 µg/mL INH showed lower MICs than MXF for the mutants, as did WQ-3810 and MXF alone (Table 3).

Subsequently, we calculated the mean fraction inhibitory concentration index (FICIm) shown in Table 4, Tables S1 and S2 of WQ-3810 and MXF with the combination of either EMB or INH. The combination of INH with WQ-3810 and MXF showed a synergistic relationship, with mean FICIms of ≤0.5, whereas most of the mean FICIms of WQ-3810 and MXF with the combination of EMB were between 0.5 and 1, indicating additive interactions. The mean FICIms of WQ-3810 and MXF with INH ranged from 0.19 to 0.71, and those of WQ-3810 and MXF with EMB ranged from 0.41 to 0.91.

Concentration ranges as follows: WQ-3810 (0.125–32 µg/mL), MXF (0.125–32 µg/mL), EMB (0.0625–128 µg/mL), and INH (0.0625–128 µg/mL). Each experiment was conducted in triplicate to confirm the reproducibility. Synergistic activity was evaluated by calculating the fraction inhibitory concentration index (FICI) and taking the cube root. The mean minimum FICI (FICIm) ≤ 0.5 is regarded as synergistic.

## 3. Discussion

Novel FQs are essential for treating MAC-PD, as cases of the disease are steadily increasing worldwide [6,7]. Furthermore, reports of FQ-resistant *M. avium* strains have been documented [17,25]. WQ-3810 is an FQ with structural modifications at the N_1_, R_7_, and R_8_ positions, which confer considerable efficacy against a range of FQ-resistant bacteria, including *M. tuberculosis* and *M. leprae* [22,23,24].

This study sought to evaluate the efficacy of WQ-3810 against *M. avium* through a supercoiling inhibitory assay, an MIC assay, and a checkerboard assay. We used recombinant DNA gyrases, including WT, and those with Ala91Val, Asp95Ala, Asp95Gly, and Asp95Tyr substitutions in GyrA, for the supercoiling inhibitory assay, given the significant association of these substitutions with FQ resistance and their impact on resistance mechanisms [17,18].

The outcomes of the supercoiling inhibitory assay indicated that WQ-3810 exhibited greater potency compared to LVX and CIP in inhibiting the activity of both WT and mutant DNA gyrases. Notably, the IC_50_ of CIP and LVX against GyrA-WT were 1.7–2.1 times greater than those of WQ-3810. Additionally, IC_50_ of LVX and CIP against GyrA-Asp95Ala, GyrA-Asp95Gly, and GyrA-Asp95Tyr were 5.9–7.4-fold, 3.3–7.5-fold, and 11–13.6-fold higher than that of WQ-3810, respectively. MXF exhibited a 0.5-fold reduction of IC_50_ in inhibitory effect against GyrA-WT in comparison to WQ-3810. However, MXF demonstrated a 1.6-fold and 1.7-fold higher IC_50_ for GyrA-Asp95Gly and GyrA-Asp95Tyr, respectively, than that of WQ-3810, indicating that WQ-3810 serves as a more efficacious FQ concerning the amino acid substitutions at position 95.

**Figure 4:**
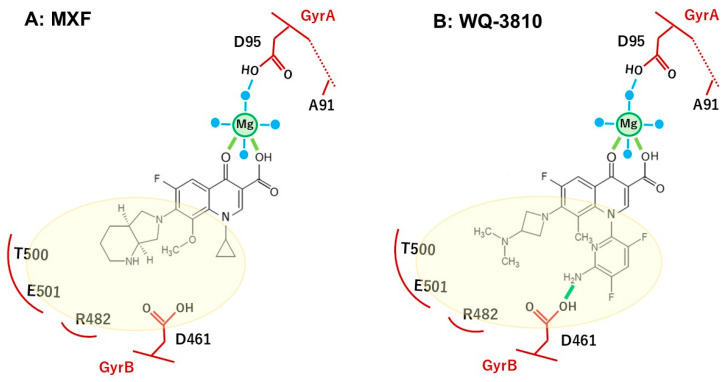
Predicted binding mode of MXF (**A**) and WQ-3810 (**B**) in the quinolone binding pocket formed by GyrA and GyrB. Mg: magnesium ion, blue dot: water molecule, blue line: water/Mg ion bridge, yellow-green line: hydrogen bond, pale-yellow circle: van der Waals interaction area. Amino acid numbers in GyrB are for the *M. tuberculosis* position.

The observed results may be elucidated by the distinct properties at positions N_1_, R_7_, and R_8_ within WQ-3810, MXF, CIP, and LVX (Figure 2 and Table 2), alongside the mode of action of FQs, which interact with the DNA-DNA gyrase complex to inhibit its function, ultimately culminating in bacterial cell death [26]. The carbonyl substituents at C_3_ and C_4_ of the quinolone ring utilize a noncatalytic Mg^2+^ ion and four water molecules to establish a water-metal ion bridge (Figure 4) [27,28]. In *E. coli*, two of the water molecules interact with the GyrA residues Ser83 and Asp87, while R_7_ interacts with GyrB [26,27,28]. Amino acid position 83 in *E. coli* corresponds to position 91 in *M. avium* and 90 *in M. tuberculosis* (Supplementary Figure S1). Since the amino acid sequence of the GyrA QRDR of *M. avium* is identical to that of *M. tuberculosis* and *M. leprae*, we referred to the results of molecular docking simulations using *M. tuberculosis* gyrase [22]. *M. avium* forms a partial water metal ion interaction with Asp95 due to the substitution of serine (Ser83) with alanine (Ala91) (Figure 4, Supplementary Figure S1) [28]. Consequently, *M avium* gyrase possesses a single amino acid for bridge anchoring, thereby resulting in weaker DNA gyrase-FQ interactions compared to other bacterial species, which can form two interactive bridges. Moreover, FQ-resistance may be exacerbated by substituting the bulky, hydrophobic valine with Ala91, as valine impairs the optimal interaction of water molecules with Mg^2+^, resulting in an IC_50_ of FQs against GyrA-Ala91Val that was 1.3-fold to 10.5-fold higher than that of the GyrA-WT (Table 1). Furthermore, the substitution of Asp95, an acidic amino acid residue, with a larger hydrophobic tyrosine blocks the binding site of water molecules with Mg^2^, resulting in Asp95Tyr exhibiting greater resistance to all FQs. Unique substituents introduced at positions N_1_ and R_7_ of the FQs can hydrophobically interact in binding to DNA gyrase, increasing the binding affinity to GyrB. Moreover, modifying the quinolones at positions N_1_, R_3_, R_6_, R_7_, and R_8_ has been shown to overcome resistance and increase the drug’s effectiveness against resistant bacteria by enhancing pharmacokinetics, reducing toxicity, and improving activity [14,15].

Considering the structural properties of the substituent at N_1_, R_7_, and R_8_ positions of the quinolone ring, we hypothesized that WQ-3810 has the potential for multiple interactions with GyrB in the FQ binding pocket, thereby enhancing efficacy (Figure 4). Specifically, the 5-amino-2,4-difluoropyridyl group at position N_1_ is significantly distorted out of the quinolone core, and this outlying conformation allows it to interact with the sites that are inaccessible to conventional FQs [21]. In fact, our previous docking simulation analysis using the modified *M. tuberculosis* gyrase FQ-binding pocket showed that WQ-3810 formed a hydrogen bond between D461 of GyrB and the amino group on its N_1_ pyridyl ring, while MXF did not have any specific binding sites (Figure 4) [22]. The bulky N_1_ residue also forms van der Waals interactions with the GyrB pocket, while the azetidinyl group at the R_7_ position engages in both hydrophobic interactions and hydrogen bonding. Additionally, the methyl group at the R_8_ position enhances the overall stability, which may contribute to WQ-3810 higher binding affinity for DNA bases, leading to a lower IC_50_ [28]. Although the piperazine group at the R_7_ position of CIP interacts with GyrB through hydrogen bonds [23], these interactions are weaker than those formed by the substituents in WQ-3810, resulting in a higher IC_50_ value for CIP. Even though MXF and CIP have a similar cyclopropyl group at the N_1_ position, MXF’s efficacy is comparable to that of WQ-3810 due to its azabicyclic group at the R_7_ position and methoxy group at the R_8_ position, which forms additional hydrogen bonds and van der Waals interactions with GyrB [23]. However, WQ-3810 demonstrates higher potency against the amino acid substituents at position 95 in GyrA, suggesting a higher binding affinity for DNA gyrase than MXF, possibly due to the hydrogen bond between the D461 of GyrB and the amino group on the N_1_ substituent of WQ-3810 (Figure 4).

We then performed a MIC assay on 11 clinical isolates and found that WQ-3810 exhibited a lower MIC than LVX and CIP, with reductions of 4 to 16-fold and 1 to 8-fold, respectively. Notably, WQ-3810’s MIC of 0.125 to 1 µg/mL was comparable to MXF’s MIC of 0.25 to 1 µg/mL against WT strains, and WQ-3810’s MIC of 4 to 8 µg/mL was half that of MXF’s MIC of 8 to 16 µg/mL for the mutant strains, demonstrating its superiority against mutant strains. The results of the supercoiling inhibitory assay indicated that mutant gyrases exhibited the lowest IC_50_s at position 95, further supporting the superiority of WQ-3810.

Since MXF-containing regimens have improved the treatment outcomes for MAC-PD [29], MXF has been suggested as a potential secondary drug [12,13,30,31] for macrolide-resistant MAC isolates or for patients who are unable to undergo macrolide therapy. The proposed breakpoints were ≤1 µg/mL, 2 µg/mL, and >4 µg/mL, indicating susceptible, intermediate resistant, and resistant, respectively, [12]. WQ-3810 demonstrated superior antimicrobial activity compared to MXF, suggesting this novel agent may be an effective replacement for MXF for MAC-PD.

Before reaching the target protein, i.e., DNA gyrase, FQs need to pass through the bacterial cell wall and adequately accumulate inside the bacterial cell to exhibit strong antimicrobial activity. However, the mycobacterial cell wall consists of a thick, lipid-rich mycolic acid, which accounts for 60% of the mycobacterial mass in *M. tuberculosis*, making it highly hydrophobic [32]. Therefore, many researchers have attempted to develop lipophilic FQs to enhance cell wall penetration [33]. A previous study indicated that the bulky azetidinyl group at the R_7_ position of WQ-3810 increases the drug’s lipophilicity by introducing an alkyl group into 7-(3-aminoazetidin-1-yl) FQ [20]. Results from this study suggest that WQ-3810 exhibits increased permeability, facilitating easy penetration due to the high lipophilic substituent at the C_7_ and C_8_ positions of the compound. Our findings show that the MIC of WQ-3810 was half that of MXF for Asp95Gly and Asp95Tyr, demonstrating superior antimicrobial activity. In contrast, another study used recombinant *M. tuberculosis* biovar. *bovis* Bacille Calmette–Guerin strains found that WQ-3810 displayed weaker antimycobacterial activity compared to MXF [23]. This discrepancy may arise from differences in intrinsic drug resistance mechanisms, such as variations in efflux pump mechanisms [34,35], distinct structural characteristics of the cell wall, and different physiological traits among the species [36].

We furthermore used the cell wall synthesis inhibitors INH and EMB in a checkerboard assay to evaluate their interaction with WQ-3810 in *M. avium* clinical isolates. These inhibitors block the synthesis of the lipid-rich mycobacterial cell wall, increasing its permeability and facilitating the passage of FQs to the DNA gyrase [9,10]. EMB inhibits arabinosyl-transferase, disrupting arabinogalactan in the cell wall [37]. INH inhibits cell wall production by preventing the synthesis of mycolic acids [38]. MXF was selected among three drugs for the checkerboard assay to compare with WQ-3810 due to its comparable results in other assays. Our findings indicate that both WQ-3810 and MXF exhibit a synergistic relationship (mean FICIm < 0.5) when used in combination with INH, while they showed additive effects (mean FICIm between 0.5 and 1) when combined with EMB, suggesting that INH enhances the permeability of *M. avium’s* cell wall to antimicrobial agents compared to EMB. However, previous studies have shown that EMB synergizes with various other drugs [23,37]. We therefore measured the MIC of WQ-3810 and MXF with the combination of 1 µg/mL of EMB and INH. For both WT and mutant strains, the MICs of both FQs were similar to or lower, when they were used alone, highlighting that WQ-3810 can be effective in combination drug therapy with other agents. Notably, when combined with either 1 µg/mL INH or EMB for mutant strains, WQ-3810 demonstrated a lower MIC than the MIC of MXF. WQ-3810’s superiority was further supported by the findings from the supercoiling inhibitory assay and MIC assay, which indicated that the mutant gyrase with a substitution at position 95 exhibited the lowest IC_50_ and MIC values. Our findings suggest that WQ-3810 is a promising therapeutic option, demonstrating higher activity against mutants compared to MXF, a second-line drug currently used for macrolide-resistant MAC isolates.

## 4. Materials and Methods

### 4.1. Recombinant M. avium Subsp. Hominissuis DNA Gyrases

Recombinant *M. avium* subsp. *hominissuis* WT and mutant DNA gyrases were used. Four mutant DNA gyrases were selected that showed single amino acid substitutions compared to WT: Ala91Val, Asp95Ala, Asp95Gly, and Asp95Tyr were selected. All the DNA gyrase used in the present study was produced in our previous study [17].

### 4.2. Clinical Isolates of M. avium Subsp. Hominissuis

Ten clinical *M. avium* subsp. *hominissuis* isolates from Keio University Hospital, Tokyo, and one from Osaka Habikino Medical Center, Osaka, were used. The isolates included 7 WT strains, 2 strains with the Asp95Gly mutation, and 2 strains with the Asp95Tyr mutation. MIC assay and Checkerboard assay were not performed for the clinical strains with Ala91Val and Asp95Ala, as these mutations were not found in the clinical isolates [17].

### 4.3. Antimicrobial Compounds and Reagents

WQ 3810 was a gift from Wakunaga Pharmaceutical Co., Ltd. (Tokyo, Japan). CIP, MXF, and LVX were purchased from LKT Laboratories, Inc. (St. Paul, MN, USA). EMB was obtained from MP Biomedicals (Santa Ana, CA, USA). The chemical structures of these four FQs are shown in Figure 1 and Table 2. Kanamycin (KAN), isoniazid (INH), and Tween 80 were purchased from Fujifilm Wako Pure Chemical Co., Ltd. (Osaka, Japan). GelRed (10,000× concentration) was bought from Biotium (San Francisco, CA, USA). Relaxed pBR322 DNA was obtained from John Innes Enterprises Ltd. (Norwich, UK). Agarose gel I was obtained from Dojindo (Kumamoto, Japan). Lambda DNA-HindIII DNA marker was obtained from New England Biolabs, Inc. (Ipswich, MA, USA). 2% Ogawa medium was obtained from Serotec Co., Ltd. (Sapporo, Japan). BD Difco Middlebrook 7H9 broth and BD BBL Middlebrook OADC Enrichment were obtained from Becton, Dickinson, and Company (Franklin Lakes, NJ, USA).

### 4.4. FQs Inhibited DNA Gyrase Supercoiling Assay

The DNA supercoiling inhibitory assay was performed using the purified recombinant DNA gyrases as described previously (Supplementary Figure S2) [17]. In brief, 30 µL of the reaction mixture was prepared containing 1× DNA gyrase reaction buffer (35 mM of tris-HCl (pH 7.5), 24 mM of KCl, 6 mM of MgCl_2_, 5 mM of DTT, 1.8 mM of spermidine, 6.5% glycerol (wt/vol), 0.36 mg/mL of BSA), 50 mM ATP, 1.5 nM relaxed pBR322 DNA, 7.5 nM Gyr A, 7.5 nM Gyr B and serially diluted WQ-3810. WQ-3810 was utilized at a concentration range of 0.125 µg/mL to 64 µg/mL. Then, the reaction mixture was incubated at 37 °C for 60 min, and the reaction was stopped by adding 8 µL of 5× dye (5% SDS, 25% glycerol, 0.25mg/mL bromophenol blue). Next, 10 µL of each reaction mixture and 5 µL of 500 ng/mL Lambda DNA-HindIII DNA marker (New England Biolabs) were loaded onto a 1% agarose I gel in 1× Tris-acetate-EDTA buffer for electrophoresis at 50 mA for 96 min. Subsequently, the gels were stained with 1× GelRed for 30 min, and the presence of a supercoiled DNA band was visualized under UV light. The amount of supercoiled DNA was quantified by measuring the intensity of the bands by using ImageJ 1.54d software (https://imagej.net/ij/download.html, accessed on 24 May 2023). The concentration of WQ-3810 that reduced DNA gyrase activity by 50% (IC_50_) was calculated with the AAT Bioquest IC_50_ calculator web tool (https://www.aatbio.com/tools/ic50-calculator, accessed on 24 May 2023). All the assays were run in triplicate on the same day to eliminate experimental bias and to confirm their reproducibility.

### 4.5. Minimum Inhibitory Concentration (MIC) Assay

The MIC assay was performed using the microdilution method in Middlebrook 7H9 medium, supplemented with 10% oleic acid-albumin-dextrose-catalase (OADC) and 0.05% Tween 80 following the protocol recommended previously [17]. Briefly, the preserved isolates were inoculated in 2% Ogawa medium, and solid culture was sub-cultured in 4 mL of 7H9 broth and incubated at 37 °C until the optical density (OD) at 590 nm reached 0.15. Next, this culture was further diluted 40 times and used for the MIC assay. The assay was performed in a 96-well round-bottom culture plate (As One Co. Ltd., Osaka, Japan). Each well contained a 200 µL mixture of 100 µL of starting culture and 100 µL of serially diluted drug in 7H9 broth. The outer wells of the plate contained 200 µL of sterile distilled water. Each plate of the MIC assay contained two drug controls with KAN at 25 µg/mL to observe growth inhibition, two growth controls without any drugs to see bacterial growth, and two medium controls without bacteria to check for contamination of the medium. For this study, WQ-3810, MXF, EMB, and INH were used. After sealing the plate with a plastic membrane, it was placed in a container with moist cotton and incubated at 37 °C for 14 days. Each experiment was conducted in triplicate to confirm the assay’s reproducibility. Bacterial growth was monitored on days 0, 1, 7, 10, and 14 by taking a picture. The MIC value, the lowest concentration of the drug at which bacterial growth was completely inhibited, was determined on day 14.

### 4.6. Checkerboard Assay

A checkerboard assay was conducted to evaluate the interaction between FQs (WQ-3810 and MXF) and cell wall synthesis inhibitors (INH and EMB) in clinical *M. avium* subsp. *hominissuis* isolates. Each agent’s fraction inhibitory concentration index (FICI) was determined in the presence of sub-inhibitory concentrations of another agent, following the protocol previously recommended [23,39]. The assay was performed in Middlebrook 7H9 broth, supplemented with 10% OADC and 0.5% Tween 80. Bacterial cultures were prepared to an OD of 0.15 at 590 nm and diluted 40-fold in Middlebrook 7H9 broth. Then 100 µL of the diluted culture was added to each well of a sterile round-bottom microtiter plate containing serially diluted concentrations of FQs (WQ-3810 or MXF) (50 µL per well) and either INH or EMB (50 µL per well). The test plates were incubated at 37 °C for 14 days, and bacterial growth was monitored by taking photos of the plates on days 0, 1, 7, 10, and 14. Each experiment was conducted in triplicate to confirm the assay’s reproducibility, and the cube root value was calculated. The FICI for each drug combination was calculated using the equation: FICI = (MIC of drug A in the presence of drug B/MIC of drug A alone) + (MIC of drug B in the presence of drug A/MIC of drug B alone). The FICIm values ≤ 0.5 were considered indicative of synergistic interaction.

### 4.7. Statistical Analysis

Statistical analysis was performed using RStudio version 2024.04.1 to compare the effects of four different FQs on inhibitory concentration (IC) values. A one-way analysis of variance (ANOVA) was used to identify any overall significant differences among the drugs. Furthermore, a post hoc Least Significant Difference (LSD) test was conducted to identify specific drug pairs with significant differences.

## 5. Conclusions

Our study assessed the effectiveness of WQ-3810, a novel FQ, against *M. avium* DNA gyrases and clinical isolates. WQ-3810 showed a stronger inhibitory effect than LVX and CIP and was comparable to or superior to MXF for both WT and mutant DNA gyrases. WQ-3810 exhibited lower MICs against clinical isolates, especially FQ-resistant isolates, than other FQs. Furthermore, WQ-3810 showed a synergistic interaction in combination with INH. Our results indicate that WQ-3810 is a promising therapeutic option, demonstrating superior efficacy compared to MXF, which is currently available as a second-line treatment for MAC-PD resistant to macrolide therapy. WQ-3810 may also be a potential treatment option for FQ-resistant *M. avium* infections.

## Figures and Tables

**Table 1:** IC_50_s (µg/mL) of fluoroquinolones against WT and mutant DNA gyrases.

	WT	Ala91Val	Asp95Ala	Asp95Gly	Asp95Tyr	Reference
WQ-3810	1.7 ± 0.1	9.9 ± 0.6	4.5 ± 1.1	7.0 ± 0.5	7.8 ± 0.6	This study
CIP	3 ± 0.8	10.2 ± 0.3	33.4 ± 7.0	53 ± 4.7	106.8 ± 4.0	Previous study [17]
MXF	0.9 ± 0.1	9.5 ± 0.2	4.2 ± 0.3	11.3 ± 3.5	13.5 ± 0.4	Previous study [17]
LVX	3.7 ± 0.4	5 ± 1.5	26.6 ± 5.9	23.3 ± 2.4	86.1 ± 6.0	Previous study [17]

**Table 2:** Structure of fluoroquinolones.

Fluoroquinolones	N_1_	R_6_	R_7_	R_8_
CIP	Cyclopropyl	F	Piperazine	H
LVX	Bridge N_1_-C_8_	F	4-Methylpiperazinyl	Bridge N_1_-C_8_
MXF	Cyclopropyl	F	Piperridino-pyrrolidinyl	CH_3_O
WQ-3810	5-Amino-2,4-difluoropyridine-2-yl	F	3-Isopropylaminoazetizine-1-yl	CH_3_

**Table 3:** Drug susceptibility of clinical isolates.

Sample ID	GyrA Mutation	MIC (µg/mL) of:						MIC (µg/mL) with 1 µg/mL EMB		MIC (µg/mL) with 1 µg/mL INH	
		LVX	CIP	MXF	WQ-3810	EMB	INH	MXF	WQ-3810	MXF	WQ-3810
Koav 3	Asp95Gly	64	32	8	4	16	4	8 (×1)	4 (×1)	4 (×0.5)	2 (×0.5)
Koav 11	Asp95Tyr	8	16	16	4	16	8	8 (×0.5)	4 (×1)	8 (×0.5)	4 (×1)
Koav 13	Asp95Gly	128	64	16	8	32	32	16 (×1)	4 (×0.5)	8 (×0.5)	4 (×0.5)
Cl-A-2	Asp95Tyr	64	32	8	4	16	4–8	4 (×0.5)	1 (×0.25)	8 (×1)	1(×0.25)
Koav 1	None (WT)	<0.5	0.5	0.25	0.125	2	8	<0.01 (×0.04)	0.01 (×0.08)	0.03 (×0.12)	0.03 (×0.24)
Koav 2	None (WT)	2	1	1	0.5	16	128	ND	ND	ND	ND
Koav 15	None (WT)	4	2	1	1	16	32	1 (×1)	1 (×1)	1(×1)	1 (×1)
Koav 19	None (WT)	2	2	0.5	0.5	16	8–16	0.5 (×1)	0.25 (×0.5)	0.13 (×0.25)	0.13 (×0.5)
Koav 20	None (WT)	<0.5	0.5	0.25	0.125	2	16	0.13–0.25 (×0.5–1)	0.13 (×1)	0.25 (×1)	0.13 (×1)
Koav 21	None (WT)	4	1	1	1	32	16	1 (×1)	1 (×1)	0.5 (×0.5)	1 (×1)
Koav 25	None (WT)	4	2	1	1	32	8	1 (×1)	0.5 (×0.5)	0.5 (×0.5)	0.5 (×0.5)
Reference		Previous study [17]	This study	This study	This study	This study	This study				

Concentration ranges as follows: LVX (0.5–128 µg/mL), CIP (0.5–128 µg/mL), MXF (0.125–32 µg/mL), WQ-3810 (0.125–32 µg/mL), EMB (0.0625–128 µg/mL), INH (0.0625–128 µg/mL), ND: not determined.

**Table 4:** Mean FICIm of WQ-3810 and MXF with cell wall inhibitors.

Sample ID	Gyr A Mutation	Mean FICIm of WQ-3810		Mean FICIm of MXF	
		with INH	with EMB	with INH	with EMB
Koav 3	Asp95Gly	0.71	0.75	0.66	0.52
Koav 11	Asp95Tyr	0.46	0.83	0.41	0.52
Koav 13	Asp95Gly	0.31	0.83	0.25	0.6
Cl-A-2	Asp95Tyr	0.53	0.66	0.40	0.41
Koav 1	None (WT)	0.45	0.62	0.27	0.5
Koav 2	None (WT)	0.25	0.91	0.31	0.71
Koav 15	None (WT)	0.33	0.71	0.38	0.66
Koav 19	None (WT)	0.45	0.86	0.19	0.71
Koav 20	None (WT)	0.25	0.66	0.41	0.81
Koav 21	None (WT)	0.41	0.75	0.31	0.55
Koav 25	None (WT)	0.39	0.75	0.49	0.75

## Data Availability

The original contributions presented in this study are included in the article/Supplementary Materials. Further inquiries can be directed to the corresponding authors.

## Supplementary Materials

The following supporting information can be downloaded at https://www.mdpi.com/article/10.3390/antibiotics14070704/s1, Table S1: Interaction between WQ-3810 and cell wall inhibitors. Table S2: Interaction between MXF and cell wall inhibitors. Figure S1: Amino acid sequence alignment of DNA gyrases QRDR from various bacterial species. Figure S2: SDS-PAGE analysis of purified recombinant *M. avium* DNA gyrase subunits.
