# Supplementary material for: Investigation of WQ-3810, a Fluoroquinolone with a High Potential Against Fluoroquinolone-Resistant Mycobacterium avium
Source: Antibiotics (Basel). 2025 Jul 14;14(7):704. doi: 10.3390/antibiotics14070704 (PMC12291913; doi:10.3390/antibiotics14070704)
Supplement: Supplementary file 1 [file antibiotics-14-00704-s001.zip › GyrA QRDR alignment supple fig S1.pptx]

## Slide 1
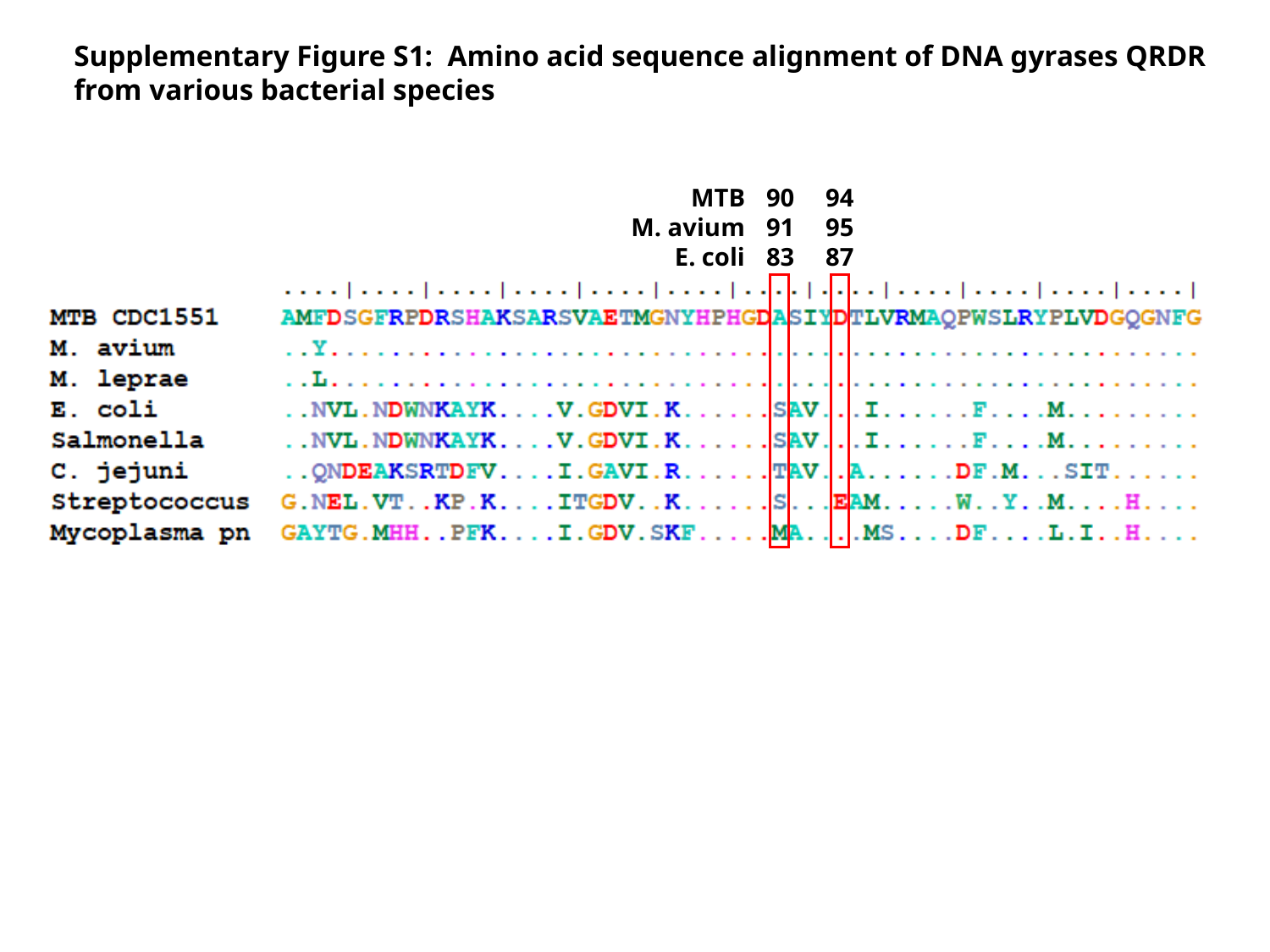

Supplementary Figure S1: Amino acid sequence alignment of DNA gyrases QRDR from various bacterial species
MTB
M. avium
E. coli
90
91
83
94
95
87
