# Supplementary material for: Investigation of WQ-3810, a Fluoroquinolone with a High Potential Against Fluoroquinolone-Resistant Mycobacterium avium
Source: Antibiotics (Basel). 2025 Jul 14;14(7):704. doi: 10.3390/antibiotics14070704 (PMC12291913; doi:10.3390/antibiotics14070704)
Supplement: Supplementary file 1 [file antibiotics-14-00704-s001.zip › supplementary figure 2.pdf]

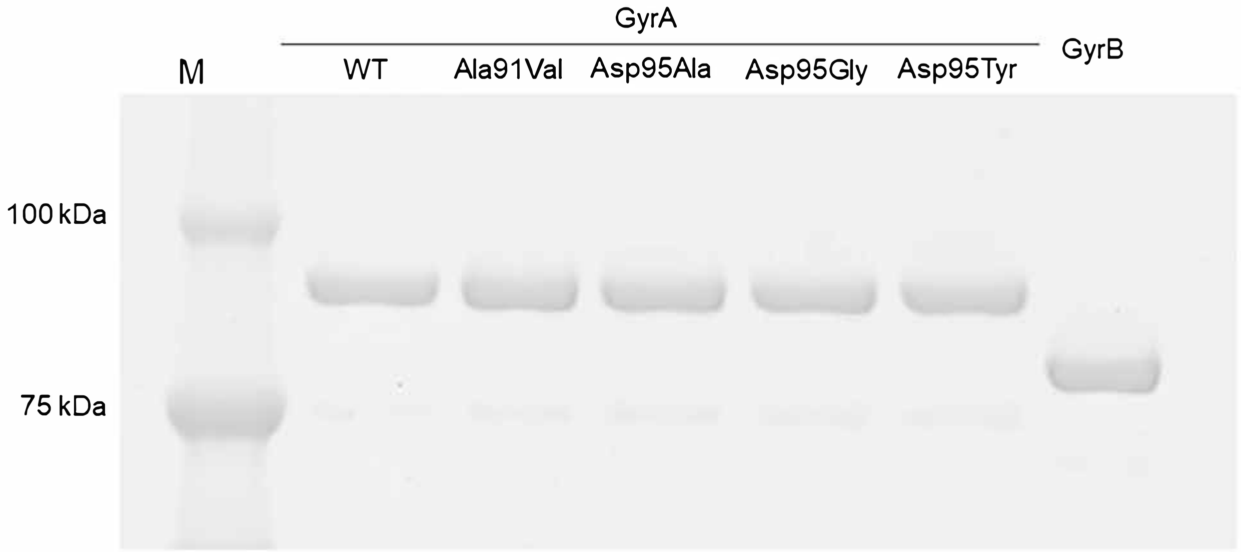

Fig. S2. SDS-PAGE analysis of purified recombinant *M. avium* DNA gyrase subunits. Three hundred ng of each recombinant DNA gyrase subunits were loaded into a SuperSep (TM) Ace, 5-20%, 17 well gel (Fujiflim Wako, Osaka, Japan) for electrophoresis. Reference [17] https://doi.org/10.1128/spectrum.05088-22
